# Quantitative Modeling of IgG N-Glycosylation Profiles from Population Data

**DOI:** 10.3390/ijms262311495

**Published:** 2025-11-27

**Authors:** Elena Kutumova, Nikita Mandrik, Ruslan Sharipov, Maja Pučić-Baković, Borna Rapčan, Yurii Aulchenko, Gordan Lauc, Fedor Kolpakov

**Affiliations:** 1 Department of Computational Biology, Sirius University of Science and Technology, 354340 Sirius, Russia; 2 Laboratory of Bioinformatics, Federal Research Center for Information and Computational Technologies, 630090 Novosibirsk, Russia; 3 Biosoft.Ru, Ltd., 630058 Novosibirsk, Russia; 4 Specialized Educational Scientific Center, Novosibirsk State University, 630090 Novosibirsk, Russia; 5 Genos Glycoscience Research Laboratory, 10000 Zagreb, Croatia; 6 Laboratory of Theoretical and Applied Functional Genomics, Novosibirsk State University, 630090 Novosibirsk, Russia; 7 Laboratory of Recombination and Segregation Analysis, Institute of Cytology and Genetics, Siberian Branch of Russian Academy of Sciences, 630090 Novosibirsk, Russia; 8 Faculty of Pharmacy and Biochemistry, University of Zagreb, 10000 Zagreb, Croatia

**Keywords:** immunoglobulin G, N-glycosylation, Golgi apparatus, rule-based modeling, BioUML

## Abstract

Glycosylation of immunoglobulin G (IgG) is a critical regulator of its functional properties. We present an original mathematical model, calibrated and validated using quantitative IgG N-glycosylation data from two independent cohorts, 915 individuals from Korčula Island and 890 individuals from Vis Island, Croatia, reported in prior studies. The datasets comprise relative glycan levels measured by ultrahigh-performance liquid chromatography (UHPLC), represented by 22 chromatographic peaks per individual. By fitting the model to these data, we estimated the total concentrations of seven key enzymes involved in glycan biosynthesis across four Golgi compartments. The model revealed an age-related decline in β-N-acetylglucosaminylglycopeptide β-1,4-galactosyltransferase (GalT) concentrations in both populations, emphasizing its essential role in driving age-dependent changes in IgG glycan profiles and underscoring its potential as a biomarker of aging.

## 1. Introduction

Asparagine (Asn) N-linked glycosylation is a fundamental and extensive post-translational modification involving the covalent attachment of an oligosaccharide (glycan) to Asn residues within polypeptide chains [1,2,3]. N-glycans significantly influence glycoprotein properties such as conformation, solubility, and antigenicity [4]. Defects in glycan biosynthesis and metabolism can lead to severe hereditary diseases in humans [5].

N-glycosylation initiates in the endoplasmic reticulum (ER) through the stepwise synthesis of a lipid-linked oligosaccharide precursor. This process starts on the cytoplasmic side of the ER membrane, where N-acetylglucosamine (GlcNAc) and mannose (Man) residues are sequentially attached to dolichol phosphate. The partially assembled glycan-lipid intermediate is subsequently flipped into the ER lumen, where it is elongated by further addition of mannose and glucose (Glc) residues, forming the mature Glc_3_Man_9_GlcNAc_2_ structure. Following this, an oligosaccharyltransferase transfers the carbohydrate chain to a nascent protein at the Asn-X-Ser/Thr site, where X represents any amino acid except proline. The resulting glycoprotein undergoes glucose trimming, chaperone-assisted folding, and is then transported to the Golgi apparatus for terminal processing and maturation [6,7,8,9].

The Golgi apparatus is composed of an ordered series of compartments: the cis-Golgi network, cis-, medial-, and trans-cisternae, followed by the trans-Golgi network [10,11]. In the cis-Golgi, carbohydrate moieties are trimmed by specific mannosidases before being transferred to the medial-Golgi for further maturation. Within the medial and trans-Golgi compartments, N-glycans undergo additional processing, including the sequential addition of GlcNAc, galactose, sialic acid, and fucose residues [6].

Extensive studies of immunoglobulin G (IgG) glycosylation in healthy individuals and patients with diverse diseases highlight the critical role and diagnostic potential of IgG glycans [12]. Aberrant IgG glycosylation is a hallmark feature of autoimmune diseases and contributes to their pathogenesis [13,14,15,16]. Moreover, IgG glycan profiles show promise as biomarkers for infectious diseases and cancer [17,18]. Within the concept of inflammaging, IgG glycans serve not only as biomarkers but also as molecular effectors that influence aging processes [19,20].

**Figure 1:**
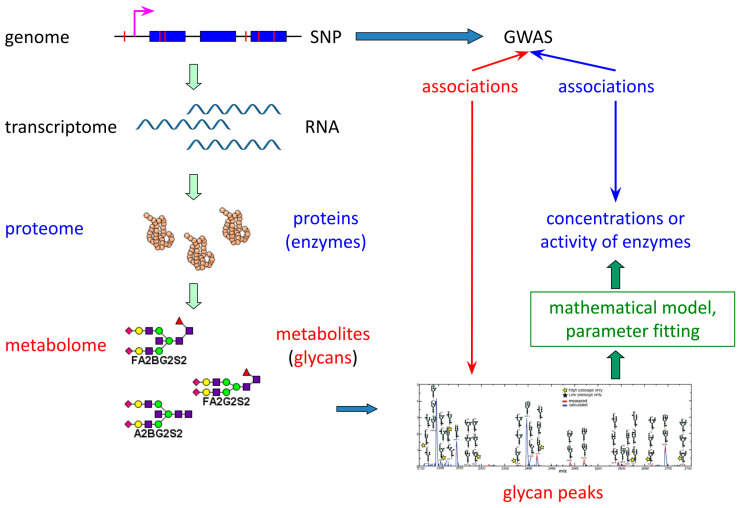
A novel approach for uncovering single nucleotide polymorphisms (SNPs) associations through mathematically inferred activities/concentrations of glycosyltransferases.

Recent population-based research of IgG N-glycosylation has demonstrated that genome-wide association studies (GWAS) of glycan levels can identify novel genetic loci involved in regulating this process [21,22,23,24,25,26]. However, linking single nucleotide polymorphisms (SNPs) to specific glycan peaks remains challenging (Figure 1). The effects of SNPs may propagate along complex biological pathways, complicating their detection by statistical methods and their subsequent interpretation.

Mathematical modeling offers a powerful means to bridge this gap by estimating the concentrations or activities of glycosylation-related enzymes as intermediate variables (Figure 1). Subsequently, SNP associations can be sought with these recovered enzyme concentrations/activities—quantities that are not independently identifiable within the model but collectively represent glycosylation regulation. This approach aims to improve the reliability of SNP identification and simplify their biological interpretation compared to direct glycan peak analysis.

In this study, we present the initial step of this strategy—recovering glycosyltransferase activities through mathematical modeling. The developed model was calibrated and validated with experimental data from two European cohorts (Korčula and Vis Island, Croatia) [27]. Furthermore, we demonstrate that the recovered glycosyltransferase activities predict biological age with an accuracy comparable to traditional glycan peak-based markers, underscoring the model’s promise for biomarker development.

## 2. Results

### 2.1. Model Construction

**Figure 2:**
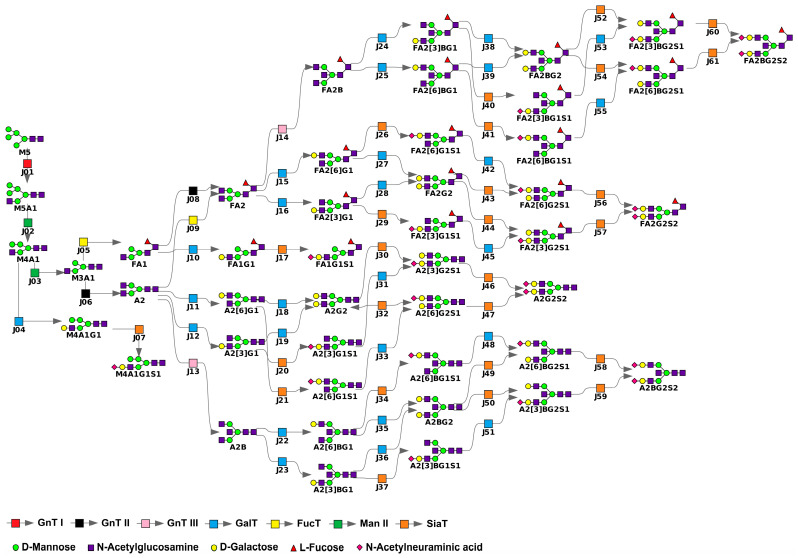
Visualization of the mathematical model of immunoglobulin G (IgG) N-glycosylation, comprising 45 glycan structures and 61 enzymatic reactions (labeled J01–J61) involved in glycan processing. To improve visual clarity, reactions associated with glycan transport between Golgi compartments are omitted. Detailed information on model parameters and components is provided in Supplementary Tables S1–S4.

We constructed a mathematical model of IgG N-glycosylation (Figure 2) by integrating data from the KEGG database [28] and extending it based on the glycan synthesis rules established by Krambeck et al. [29,30,31]. These rules comprehensively define the human N-linked glycosylation reaction network. Our model incorporates reactions involving glycans identified by mass spectrometry in the study by Pučić et al. [27], along with essential intermediate steps. Glycan structures used in the model are detailed in Supplementary Table S1, following the standards described by Banin et al. [32]. The enzymatic reactions are catalyzed by GnT I, GnT II, GnT III, FucT, Man II, GalT, and SiaT (Supplementary Table S2), each characterized as follows:

1. GnT I, GnT II, and GnT III mediate the attachment of a single N-acetylglucosamine via β1,2-linkage (GnT I, GnT II) or β1,4-linkage (GnT III) to the α1,3-linked (GnT I), α1,6-linked (GnT II), or β-linked (GnT III) mannose residue.
2. Man II catalyzes the cleavage of α1,3- and α1,6-linked mannose residues.
3. FucT transfers a fucose residue to the innermost N-acetylglucosamine of N-glycans via α1,6-linkage.
4. GalT adds galactose to terminal N-acetylglucosamine through β1,4-linkage.
5. SiaT facilitates the attachment of N-acetylneuraminic acid to galactose via α2,3- and α2,6-linkages.

Reactions and parameters of the model are listed in Supplementary Tables S3 and S4, respectively. The model starts with the M5 structure containing five mannose residues (Figure 2, Supplementary Table S1). We excluded the reaction chain responsible for generating M5, which initiates in the ER and continues through the cis-Golgi network and cis-Golgi cisternae, due to insufficient experimental data for kinetic parameters in this segment [30,33]. Consequently, the model encompasses reactions occurring in four Golgi compartments: cis-, medial-, and trans-cisternae, along with the trans-Golgi network. Previously measured chromatographic glycan peaks (22 in total, denoted as $GP_{i}$, i=1,…,24, i≠3, 20), representing specific glycan combinations [27], are integrated via equations provided in Supplementary Table S5. Detailed mathematical formulation of the model construction is provided in the Materials and Methods section. The model source file can be accessed online (refer to the Data Availability section).

### 2.2. Model Calibration

Model parameters were categorized into two groups: common parameters, assumed constant across all subjects in the experimental population, and individual parameters, specific to each subject. We designated all kinetic rate constants and distribution coefficients of seven enzymes across four Golgi compartments as common parameters. Additionally, the total concentration of Man II was treated as common, since glycans influenced by this enzyme were excluded from the $GP_{i}$ calculation (Supplementary Table S5). Model calibration involved estimating the common parameters using experimental data from the Korčula cohort. A detailed description of the optimization process follows, with a summary presented in Table 1.

Initial values for the rate parameters kf and Km in Equation (1) of the Materials and Methods section, used to calculate glycan generation rates, were adopted from the model by Bennun et al. [31]. To account for potential variations in these parameters arising from different glycan structures for the same enzyme [29,30,31], correction factors for kf  were introduced to enhance agreement with experimental data [27]. These factors were initially applied across all 61 enzymatic reactions and, together with total enzyme concentrations, optimized individually for each subject in the population. Subsequently, the number of correction factors was reduced to 22 by grouping those associated with reactions producing glycans with similar structures, provided this consolidation did not significantly impact the optimization objective function (4). The median values of the resulting parameters across the population were then fixed and listed in Supplementary Table S4.

In the second optimization stage, total enzyme concentrations and their distribution coefficients across Golgi compartments were incorporated into the fitting process. Distribution parameters were gradually removed from optimization and replaced either with values from the Krambeck model [30] or with median values when the Krambeck values caused substantial deterioration in the objective function. The final optimized distribution coefficients are listed in Supplementary Table S2. The total concentration of Man II was fixed at its median value.

### 2.3. Model Personalization for the Korčula Cohort

**Figure 3:**
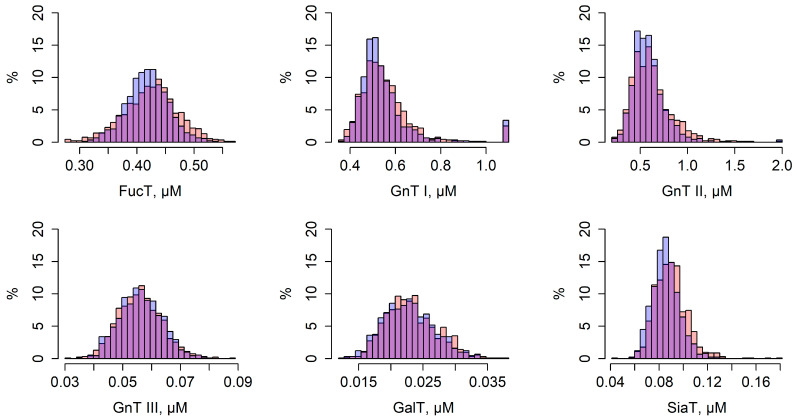
Distribution of total enzyme concentrations estimated for individuals from Korčula (red, *n* = 915) and Vis (blue, *n* = 890) populations based on the IgG N-glycosylation model. The overlapping regions are shown in purple. The *Y*-axis represents the percentage of individuals.

**Figure 4:**
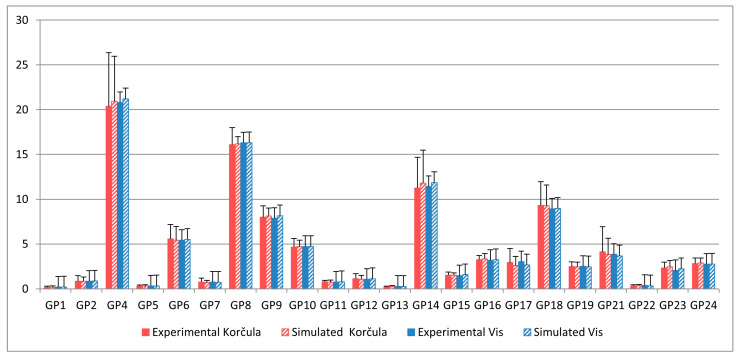
Comparison of experimental and simulated glycan peak values (%) in the Korčula (*n* = 915) and Vis (*n* = 890) populations based on data from Pučić et al. [27]. The data are presented as the mean ± SD.

Personalization of the model involved fitting six individual parameters representing total enzyme concentrations—GnT I, GnT II, GnT III, GalT, FucT, and SiaT—based on 22 individual glycan peak values for each subject in the experimental cohort (Supplementary Table S5). Figure 3 shows the distribution of these optimized values. Notably, the total concentration of GnT I reached the upper bound of its search range (1.1 μM) in some individuals. This occurred because this parameter directly affects the concentration of glycan M5, which, together with FA2, determines peak $GP_{5}$. Moreover, peak $GP_{4}$ depends exclusively on the concentration of FA2. As a result, individuals with low $GP_{5}$ but sufficiently high $GP_{4}$ drove the total GnT I level to its maximum allowed value. The mean relative deviation of simulated glycan peaks from their experimental values, calculated using Equation (5), was 17.54%. A comparison of results for all glycan peaks is shown in Figure 4.

The Mann–Whitney test identified highly significant differences (*p*-values < 10^−4^) in glycan peaks $GP_{13}$, $GP_{16}$, and $GP_{22}$ between experimental [27] and simulated data (Supplementary Table S6). This indicates that varying only six parameters in the model was insufficient to accurately reproduce the profiles of all 22 glycan peaks, underscoring the intricate regulation of glycan patterns. To preserve result clarity and interpretability, we intentionally refrained from increasing the number of variable parameters by incorporating more complex and less interpretable variability in reaction rate constants or enzyme concentration distributions within the Golgi apparatus across the population. This strategy ensured that the specific influence of enzyme concentrations remained clear, avoiding confounding factors that could mask their role in shaping glycan profiles.

### 2.4. Identifiability and Sensitivity Analysis of the Model

The identifiability analysis of personal parameters in the IgG N-glycosylation model for 915 individuals from the Korčula population showed that total concentrations of FucT, GalT, and SiaT enzymes were identifiable in all subjects. In contrast, total concentrations of GnT I, GnT II, and GnT III were partially unidentifiable in 108 (11.8%), 49 (5.4%), and 1 (0.1%) individuals, while remaining identifiable in others. Supplementary Figure S1 provides examples comparing parameter identifiability between subjects with fully identifiable enzyme sets and those with partial identifiability. These findings suggest that although most enzyme concentrations were reliably estimated, GnT I and GnT II levels in some individuals exhibited saturation, meaning further increases in enzyme concentration no longer substantially improved the model’s fit to the experimental data.

To investigate how variations in enzyme biochemical activity affect glycan peak profiles, we conducted a global Sobol sensitivity analysis using ±50% ranges around the median total enzyme concentrations estimated for the Korčula cohort (Supplementary Table S7). The Sobol total-effect indices (Supplementary Table S8) revealed that specific enzymes serve as the primary drivers of variability for individual glycan peaks—for instance, GnT I influenced $GP_{5}$; GnT II affected $GP_{1}$; GnT III regulated $GP_{10}$; SiaT controlled $GP_{23}$; GalT impacted $GP_{4}$, $GP_{6}$, $GP_{14}$, and $GP_{18}$; and FucT contributed to variability in $GP_{2}$, $GP_{7}$, $GP_{9}$, $GP_{12}$, $GP_{17}$, and $GP_{21}$. Other enzymes exhibited considerably smaller or negligible effects on these peaks. Meanwhile, several glycan peaks, including $GP_{8}$, $GP_{11}$, $GP_{13}$, $GP_{15}$, $GP_{16}$, $GP_{19}$, $GP_{22}$, and $GP_{24}$, showed moderate sensitivity to two or more enzymes such as FucT, GnT III, GalT, and SiaT, illustrating a more distributed enzymatic control involving interaction effects. First-order Sobol indices further revealed the individual influence of each enzyme on glycan variability. Thus, this analysis highlighted both enzyme-specific and combinatorial regulatory patterns shaping the glycan peak profiles.

In addition, we calculated local sensitivities of glycan peaks to median total enzyme concentrations, as well as to non-zero enzyme concentrations in each Golgi compartment—derived from total enzyme levels using the percentage distribution provided in Supplementary Table S2. The results, summarized in Supplementary Tables S9 and S10, demonstrated that glycan peak sensitivities vary according to enzyme distribution across Golgi compartments. Sensitivity to GnT I concentration was highest in the first Golgi compartment, highlighting its critical functional role there. FucT showed predominant influence in the second compartment, while GalT and SiaT exerted the strongest effects in the fourth. For GnT II and GnT III, no single compartment dominated, reflecting their overlapping activities across multiple compartments and enabling dynamic modulation of glycan structures through successive processing steps. Overall, this analysis underscores compartment-specific enzyme effects on glycosylation, suggesting that alterations in enzyme localization or activity within distinct Golgi compartments can differentially shape glycan processing patterns.

### 2.5. Model Validation with Vis Cohort Data

Model validation involved evaluating the feasibility of effective personalization on an experimental cohort independent from the data used to calibrate the common model parameters. For this, we utilized the Vis cohort. The mean relative deviation of simulated glycan peaks from their experimental values following model personalization with Vis was 16.20%, comparable to that of the Korčula cohort, indicating successful validation. The distribution of personal parameter values and the comparison between experimental and simulated glycan peaks for Vis are shown in Figure 3 and Figure 4, respectively.

### 2.6. Statistical Associations Between Modeled Enzyme Concentrations and Individual Experimental Parameters in the Populations

**Figure 5:**
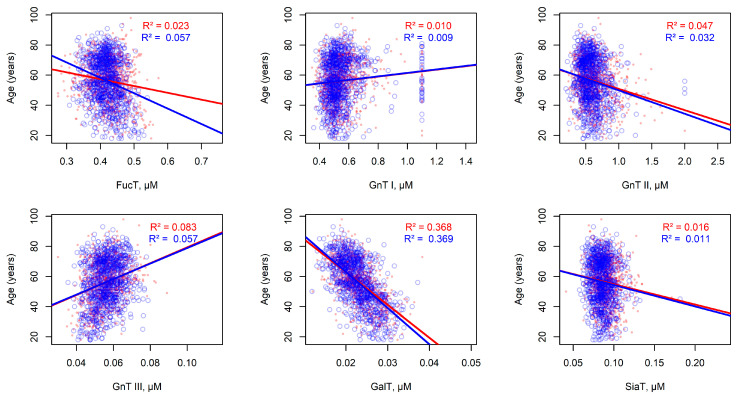
Linear regression analysis of the relationship between age and estimated total enzyme concentrations in individuals from Korčula (red, *n* = 915) and Vis (blue, *n* = 890). Each panel shows the regression lines along with the corresponding *R*^2^ values from simple linear regression using ordinary least squares.

Since human age can be predicted from peak glycan levels [34], we investigated the relationship between individual age—which was not considered during parameter optimization—and the total enzyme concentrations estimated by the model for the Korčula and Vis populations. Linear regression analysis revealed that GalT showed the strongest correlation with age in both cohorts (Figure 5). Adjusted *p*-values, corrected using Bonferroni and Benjamini–Hochberg methods, confirmed the statistical significance of this relationship (Supplementary Table S11).

Further analysis combining linear regression with leave-one-subject-out cross-validation demonstrated that GalT was the most informative enzyme predictor of age, exhibiting the lowest root mean square prediction errors: approximately 128 for Korčula and 157 for Vis cohorts. These corresponded to average age prediction errors of about 11.3 and 12.5 years, respectively (Supplementary Table S11).

**Figure 6:**
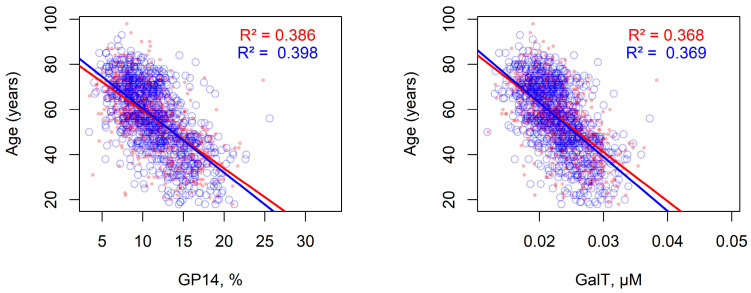
Comparison of linear regression models relating experimental $GP_{14}$ levels and simulated GalT concentrations to individual age in the Korčula (red, *n* = 915) and Vis (blue, *n* = 890) cohorts.

GalT concentration was strongly positively correlated with glycan peaks $GP_{14}$ and $GP_{18}$, as these peaks represent glycan products synthesized by GalT-catalyzed reactions: FA2G2 ($GP_{14}$) and A23BG2S1, A26BG2S1, FA23G2S1, and FA26G2S1 ($GP_{18}$). Conversely, GalT concentration exhibited a strong negative correlation with $GP_{4}$, a peak calculated from FA2, the substrate of GalT (Supplementary Tables S3, S5 and S12). Among correlations between experimental peak values [27] and age, $GP_{14}$ exhibited the highest absolute correlation (Supplementary Table S13). Comparing linear regression models for $GP_{14}$ and GalT demonstrated that their explained age-related variance (*R*^2^ values) was very similar, with GalT’s slightly lower (Figure 6).

Collectively, these findings suggest that age-related changes in IgG glycosylation are primarily driven by alterations in GalT enzyme concentration, highlighting its key role in modulating the glycosylation profile across the human lifespan. Furthermore, the transition from experimental glycan peaks to model parameters incurs only a slight reduction in prediction accuracy.

## 3. Discussion

Proteomic pathways reflect dynamic changes in functional biomolecules that drive biological processes [35]. Metabolomic data, on the other hand, capture the downstream metabolic consequences of these activities [36,37]. Thus, proteomics offers a clearer understanding of how cells function and respond to diseases by explaining mechanisms that cannot be inferred from metabolite levels alone. Although metabolic enzymes usually show coordinated expression patterns that are partly seen in metabolite profiles, the levels of metabolites are generally more variable and affected by systemic and environmental factors [38,39]. Therefore, proteomics provides a more direct connection to cellular control points and regulatory pathways, enabling more precise elucidation of molecular mechanisms [40].

In silico modeling of the glycome represents a significant advance in glycobiology, enabling efficient analysis, predictive capabilities, and mechanistic understanding of complex carbohydrate structures. This approach supports diverse applications such as disease research, drug development, and vaccine design. Recent comprehensive reviews have been provided by Akune-Taylor and colleagues [41]. Among the modern glycan models complementing our N-glycan biosynthesis framework, we highlight the O-linked glycan model developed by Kouka et al., which—like ours—is also built upon the work of Krambeck et al. [42]. This model represents an important step forward in understanding the formation of this class of glycans.

Our IgG N-glycosylation model based on 1805 European individuals (915 from Korčula and 890 from Vis Islands) showed that GalT concentrations decline with age. This additionally confirms the enzyme’s important role in age-related glycan changes and indicates it could serve as a biomarker of aging. This finding aligns with studies linking altered galactosylation patterns to aging and age-related diseases [19,34,43,44,45,46], suggesting that reduced GalT levels or activity may contribute to these glycomic shifts. In particular, galactosylation tends to decrease with age and non-galactosylated glycopeptides are more common in older individuals. A slight decrease in IgG galactosylation in middle age is considered an early marker of longer lifespan [44]. It is important to note, B-cell-specific ablation of B4GALT1 (GalT) in mice down-regulated serum IgG galactosylation level and made most age-related serum IgG glycans no longer change with age [45]. Further investigation into GalT functions could yield valuable insights into the biology of aging and support the development of targeted therapies to mitigate age-associated diseases.

### 3.1. Future Perspectives

Mathematical modeling of the IgG N-glycosylation process facilitated the transition from metabolomic analysis—focused on experimental chromatographic peak concentrations—to proteomic analysis, which examines the concentrations of enzymes catalyzing these reactions. This transition enables the integration of experimental data on individual gene mutations to refine the model and deepen our understanding of IgG functions in the human body. Additionally, advancing the model within the framework of nonlinear mixed-effects theory [47] presents a promising avenue for future research.

### 3.2. Limitations of the Study

When fitting the profiles of 22 experimental glycan peaks using six parameters representing total enzyme concentrations, we observed statistically significant deviations in three specific peaks. To maintain clarity and ensure unambiguous interpretation of effects directly related to enzyme concentrations, we deliberately refrained from introducing additional parameters in the optimization process. However, fully capturing the complexity of IgG N-glycosylation likely requires a broader modeling framework that includes regulatory mechanisms beyond enzyme levels alone. These factors may involve variability in enzyme localization [48,49], differential accessibility of glycosylation sites on the IgG molecule [50], and dynamic changes in Golgi compartmentalization [51]. Incorporating such elements into future models could improve the accuracy of simulated glycan profiles, enabling closer correspondence with experimental data and providing a more comprehensive understanding of the multifaceted regulation governing IgG glycosylation.

Additionally, the dataset used for the modeling was obtained exclusively from a limited European cohort (Korčula and Vis Islands, Croatia), which for obvious reasons may not reflect the full scope of pan-European population variability in IgG glycosylation.

## 4. Materials and Methods

### 4.1. Mathematical Formalism of the Model

The IgG N-glycosylation model comprises m=45 glycans denoted as $S_{1},\ldots ,S_{m}$ (Supplementary Table S1), with time-dependent concentrations $C^{k}\left(t\right)=\left(C_{1}^{k}\left(t\right),\ldots ,C_{m}^{k}\left(t\right)\right)$ across four Golgi compartments k=1,…,4. It also includes seven enzymes, $E_{1},\ldots ,E_{7}$, whose concentrations $U^{k}=\left(U_{1}^{k},\ldots ,U_{7}^{k}\right)$ are constant within each compartment (Supplementary Table S2).

For a glycan $S_{i}$ with concentration $C_{i}^{k}\left(t\right)$ and an enzyme $E_{j}$ with concentration $U_{j}^{k}$ in a given Golgi compartment k, the glycosylation reaction $E_{j}+S_{i}\to E_{j}+S_{h}$, which generates glycan $S_{h}$, proceeds at the rate

(1) $$v\left(C_{i}^{k}\left(t\right),U_{j}^{k}\right)=\frac{kf\cdot U_{j}^{k}\cdot C_{i}^{k}\left(t\right)}{Km}\cdot {\left(1+\sum_{l}\frac{C_{l}^{k}\left(t\right)}{{Km}_{l}}\right)}^{-1},$$

where kf and Km represent kinetic constants, and $C_{l}^{k}\left(t\right)$ are concentrations of substrates $S_{l}$ involved in all reactions catalyzed by $E_{j}$ with corresponding constants ${Km}_{l}$ [29].

The model incorporates n=61 enzymatic reactions (Supplementary Table S3) and 37 kinetic parameters (Supplementary Table S4). Reaction rates are computed as $v\left(C^{k}\left(t\right),U^{k}\right)=\left(v_{1}\left(C^{k}\left(t\right),U^{k}\right),\ldots ,v_{n}\left(C^{k}\left(t\right),U^{k}\right)\right)$ according to Equation (1), and are complemented by m irreversible glycan transport reactions between Golgi compartments. The average glycan residence time in the Golgi apparatus approximates 40 min [52]. Assuming equal volumes for all compartments, the residence time per compartment is estimated as τ = 10 min.

Under well-mixed conditions and in the absence of external influences, glycan transport between compartments is described by [29]:

(2) $$\mu \left(C^{k}\left(t\right)\right)=\frac{C^{k-1}\left(t\right)-C^{k}\left(t\right)}{\tau },\;\;k\;=\;1,\ldots ,4,$$

where $C^{0}\left(t\right)=C^{1}\left(0\right)$ represents the initial glycan concentrations in the first compartment.

The temporal dynamics of the model are governed by the system of ordinary differential equations:

(3) $$\frac{dC^{k}\left(t\right)}{dt}=N\cdot v\left(C^{k}\left(t\right),U^{k}\right)+\mu \left(C^{k}\left(t\right)\right),\;\;k\;=\;1,\ldots ,4,$$

where N is the stoichiometric matrix of dimension n×m. Initial conditions assume zero concentrations for all glycans in all compartments except for glycan M5 containing five D-mannose residues (Figure 2) in the first compartment, which is set at 1000 µM.

A steady state $\hat{C^{k}}$ for compartment k is defined by

$$N\cdot v\left(\hat{C^{k}},U^{k}\right)+\mu \left(\hat{C^{k}}\right)=0,\;\underset{t\to \infty }{\lim}C_{i}^{k}\left(t\right)=\hat{C_{i}^{k}}.$$

Steady-state glycan concentrations in the fourth compartment correspond to the steady-state solution of $C^{4}\left(t\right)$. To determine these values while accounting for Equation (2), the algebraic system

$$N\cdot v\left(\hat{C^{k}},U^{k}\right)+\frac{\hat{C^{k-1}}-\hat{C^{k}}}{\tau }=0$$

was solved sequentially. The subsystem for k=1 was solved independently for $\hat{C^{1}}$, then substituted into the equations for k=2, and so forth until k=4. This approach assumes that transitions between compartments occur only after glycan concentrations reach dynamic equilibrium. Model estimates indicate that steady state is achieved within approximately 100 min per compartment. Under these assumptions, system (3) can be reformulated as

$$\frac{dC\left(t\right)}{dt}=N\cdot v\left(C\left(t\right),U\right)+\frac{\hat{C}\left(t\right)-C\left(t\right)}{\tau },$$

with the interpretation that, in the first compartment (cis-cistern), $t\in \left[0,100\right]$ and $\hat{C}\left(t\right)=C^{1}\left(0\right)$, while enzyme concentrations correspond to fractional values indicated in column «I» of Supplementary Table S2 relative to total enzyme levels. Over the next 100 min, $C\left(t\right)$ converges toward the steady state $\hat{C^{1}}$. Enzyme concentrations then switch instantaneously to values derived from column «II», with $\hat{C}(t)=\hat{C^{1}}$ for $t\in \left[100,200\right]$. This discrete change models glycan localization in the medial-cistern, with analogous events describing transport to the trans-cisternae and trans-Golgi network compartments.

### 4.2. Experimental Data

To calibrate and validate the model, we used experimental data from populations of 915 and 890 individuals, aged 18–100 years, living on Korčula and Vis Islands (Croatia), respectively (Supplementary Figure S2). These datasets were previously published [27] and include measurements of relative glycan abundances determined as follows: IgG was purified from blood plasma of individuals, after which the N-glycans attached to IgG were released and fluorescently labeled. Using ultrahigh-performance liquid chromatography (UHPLC) with fluorescence detection, 24 chromatographic peaks $GP_{i}$, (i=1,…,24), were separated. Each peak was further analyzed by mass spectrometry to characterize the glycan structures present. Glycan quantification was obtained by measuring the areas under the respective UHPLC chromatogram peaks.

Peak $GP_{3}$ showed low intensity and high error due to contamination and was excluded from further analysis. Additionally, glycan structures in peak $GP_{20}$ could not be identified, leading to its removal from the investigation. The equations for calculating $GP_{i}$ (for i=1,…,24, excluding i=3, 20) were determined from the study by Pučić et al. [27] and are provided in Supplementary Table S5.

### 4.3. Parameter Estimation

Parameter fitting was performed by minimizing the objective function defined as the sum of normalized squared differences between the simulated (${GP}_{i}$) and experimental (${GP}_{i}^{exp}$) steady-state values of all glycan peaks [53]:

(4) $$\phi \left(GP\right)=\sum_{i}\omega_{i}\cdot {\left({GP}_{i}-{GP}_{i}^{exp}\right)}^{2},\;\omega_{i}=\frac{\min{GP}_{j}^{exp}}{{GP}_{i}^{exp}},\;i,\;j=1,\ldots ,24,\;i,\;j\neq 3,20,$$

where GP represents the set of all glycan peaks, and the weighting factors $\omega_{i}$ normalize each term to ensure equal contribution from all ${GP}_{i}$ values during fitting.

Analysis of experimental data [27] showed that the sum of the glycan peaks ${GP}_{i}$ ($GP_{peaks}$) for each of the 915 individuals ranges from 96.5% to 99.9%. This indicates that glycans outside these peaks constitute no more than 3.5% of total concentration. Accordingly, the following constraint was imposed on the sum of concentrations $GP_{all}$ of all modeled glycans:

$$g\left(GP\right)=0.965-\frac{{GP}_{peaks}}{{GP}_{all}}\leq 0,$$

and the associated penalty function incorporated in the fitting procedure was defined as:

$$\psi \left(GP\right)={max\left\{0,g\left(GP\right)\right\}}^{2}.$$

To evaluate the mean relative deviation of simulated glycan peaks from their experimental values, the following metric was used:

(5) $$D\left(GP\right)=\frac{1}{915}\cdot \frac{1}{22}\cdot \sum_{i}\sum_{j}\frac{\left|{GP}_{j}-{GP}_{j}^{exp}\right|}{{GP}_{j}^{exp}}\cdot 100\%,\;i=1,\;\ldots 915,\;j=1,\ldots ,24,\;j\neq 3,20,$$

where 22 is the number of glycan peaks included in the calculation, and 915 is the number of individuals in the experimental population.

### 4.4. Parameter Identifiability

To assess parameter identifiability, we employed the numerical method by Raue et al. [54,55], which quantifies the sensitivity of the objective function (4) to variations in each fitting parameter. This method fixes one parameter at a time, removing it from optimization, and systematically adjusts its value incrementally above and below the baseline. By analyzing the resulting changes in the objective function, which reflects the quality of fit to experimental data, we classify parameters as follows: identifiable if deviations in both directions cause a significant increase in the objective function; partially identifiable if the increase occurs in only one direction; and unidentifiable if changes produce little or no effect, indicating insufficient data to estimate the parameter reliably.

### 4.5. Global Sensitivity Analysis

Sobol sensitivity analysis was performed to quantify the contribution of total enzyme concentrations to the variance of twenty-two glycan peaks. Each enzyme concentration varied uniformly within ±50% of its estimated median value.

The first-order (main effect) Sobol index $S_{j}$ and total-effect Sobol index ${ST}_{j}$ for the model input *j* were calculated for each output using the following equations [56]:

$$S_{j}=\frac{1}{Var\left(Y\right)}\cdot \frac{1}{N}\sum_{i=1}^{N}Y_{2}^{i}\cdot \left(Y_{sj}^{i}\cdot Y_{1}^{i}\right),\;ST_{j}=\frac{1}{Var\left(Y\right)}\cdot \frac{1}{2N}\sum_{i=1}^{N}{\left(Y_{1}^{i}-Y_{sj}^{i}\right)}^{2},$$

where Var(Y) is the variance of the model output Y over the sample set; $Y_{1}^{i}$ and $Y_{2}^{i}$ are model outputs for the *i*th sample from the two independent input matrices, and $Y_{sj}^{i}$ is the output from the mixed matrix where only parameter *j* is swapped. Simulations were performed with a sample size of N = 10,000. Outputs exhibiting negligible variance were excluded to avoid numerical instability.

### 4.6. Local Sensitivity Analysis

To assess the local sensitivity of the simulation results to changes in model parameters, we calculated their relative sensitivity coefficients (SS) following Rabitz et al. [57]:

$$SS=\frac{C_{SS}\left(\alpha +∆\alpha \right)-C_{SS}\left(\alpha \right)}{∆\alpha }\cdot \frac{\alpha }{C_{SS}\left(\alpha \right)},$$

where α is the initial parameter value, Δα is the perturbation applied, and $C_{ss}\left(\alpha \right)$ and $C_{ss}\left(\alpha +\Delta \alpha \right)$ are the simulated values of the variable using the initial and perturbed parameter values, respectively.

### 4.7. Computational Methods and Software

Modeling was carried out using BioUML software (Biosoft.ru, Ltd., Novosibirsk, Russia; https://sirius-web.org/bioumlweb/; accessed on 23 November 2025) [58,59], with the model encoded in the Antimony language [60]. Simulations were performed using the JVODE solver, a Java-based implementation of the CVODE solver [61], integrated within BioUML. Parameter estimation employed a cellular genetic algorithm [62] available in BioUML, which also integrates identifiability analysis [54,55] and Sobol global sensitivity analysis [56]. Statistical calculations were performed using R (version 4.5.2, R Foundation, Vienna, Austria; https://www.r-project.org; accessed on 23 November 2025).

## Figures and Tables

**Table 1:** The sources of all common parameters.

	Common Parameters	Sources
Kinetic reaction rate constants (Supplementary Table S4)	15 *kf* and *Km* parameters for Equation (1)	[31]
	22 correction factors for *kf*	Estimated median values for the Korčula cohort
Distribution coefficients for total enzyme concentrations across Golgi compartments (Supplementary Table S2)	23 coefficients: 4 per enzyme for GnT II, GnT III, Man II, GalT, and SiaT; plus 2 for GnT I in Golgi compartments III and IV; and 1 for FucT in the final Golgi compartment	[31]
	5 coefficients: 3 for FucT across the first three Golgi compartments, plus 2 for GnT I in the first two compartments	Estimated median values for the Korčula cohort
Total enzyme concentrations	Total Man II concentration	An estimated median value for the Korčula cohort

## Data Availability

The original model source files are publicly available in the online repository: https://gitlab.sirius-web.org/virtual-cell/igg-n-glycosylation (accessed on 23 November 2025).

## Supplementary Materials

The following supporting information can be downloaded at: https://www.mdpi.com/article/10.3390/ijms262311495/s1.

## Abbreviations

The following abbreviations are used in this manuscript:

Asn	Asparagine
ER	Endoplasmic reticulum
Glc	Glucose
GlcNAc	N-acetylglucosamine
GWAS	Genome-wide association studies
IgG	Immunoglobulin G
KEGG	Kyoto encyclopedia of genes and genomes
Man	Mannose
SD	Standard deviation
SNP	Single nucleotide polymorphism
UHPLC	Ultrahigh-performance liquid chromatography
