# Supplementary material for: Quantitative Modeling of IgG N-Glycosylation Profiles from Population Data
Source: Int J Mol Sci. 2025 Nov 27;26(23):11495. doi: 10.3390/ijms262311495 (PMC12692266; doi:10.3390/ijms262311495)
Supplement: Supplementary file 1 [file ijms-26-11495-s001.zip › ijms-3976321-supplementary.pdf]

Supplementary File – Model Description and Analysis

Quantitative Modeling of IgG N-Glycosylation Profiles from Population Data

Elena Kutumova*, Nikita Mandrik, Ruslan Sharipov, Maja Pučić-Baković, Borna Rapčan, Yurii Aulchenko, Gordan Lauc, Fedor Kolpakov

***Corresponding Author:** elena.kutumova@biouml.org

Table S1. List of glycan structures of the IgG N-glycosylation model

№	Glycans	Glycan structures [32]	UHPLC peaks	KEGG ID
**01**	A2	GNb2Ma3(GNb2Ma6)Mb4GNb4GN	GP_2	G00015
**02**	A2[3]BG1	Ab4GNb2Ma3(GNb4)(GNb2Ma6)Mb4GNb4GN	GP_8	–
**03**	A2[3]BG1S1	NNa3Ab4GNb2Ma3(GNb4)(GNb2Ma6)Mb4GNb4GN	GP_{16}	–
**04**	A2[3]BG2S1	NNa3Ab4GNb2Ma3(GNb4)(Ab4GNb2Ma6)Mb4GNb4GN	GP_{18}	–
**05**	A2[3]G1	Ab4GNb2Ma3(GNb2Ma6)Mb4GNb4GN	GP_7	–
**06**	A2[3]G1S1	NNa3Ab4GNb2Ma3(GNb2Ma6)Mb4GNb4GN	GP_{15}	–
**07**	A2[3]G2S1	NNa3Ab4GNb2Ma3(Ab4GNb2Ma6)Mb4GNb4GN	GP_{17}	G00393
**08**	A2[6]BG1	GNb2Ma3(GNb4)(Ab4GNb2Ma6)Mb4GNb4GN	GP_8	–
**09**	A2[6]BG1S1	GNb2Ma3(GNb4)(NNa3Ab4GNb2Ma6)Mb4GNb4GN	GP_{16}	–
**10**	A2[6]BG2S1	Ab4GNb2Ma3(GNb4)(NNa3Ab4GNb2Ma6)Mb4GNb4GN	GP_{18}	–
**11**	A2[6]G1	GNb2Ma3(Ab4GNb2Ma6)Mb4GNb4GN	GP_6	–
**12**	A2[6]G1S1	GNb2Ma3(NNa3Ab4GNb2Ma6)Mb4GNb4GN	GP_{15}	–
**13**	A2[6]G2S1	Ab4GNb2Ma3(NNa3Ab4GNb2Ma6)Mb4GNb4GN	GP_{17}	G00837
**14**	A2B	GNb2Ma3(GNb4)(GNb2Ma6)Mb4GNb4GN	GP_3	G00019
**15**	A2BG2	Ab4GNb2Ma3(GNb4)(Ab4GNb2Ma6)Mb4GNb4GN	GP_{13}	–
**16**	A2BG2S2	NNa3Ab4GNb2Ma3(GNb4)(NNa3Ab4GNb2Ma6)Mb4GNb4GN	GP_{22}	–
**17**	A2G2	Ab4GNb2Ma3(Ab4GNb2Ma6)Mb4GNb4GN	GP_{12}	–
**18**	A2G2S2	NNa3Ab4GNb2Ma3(NNa3Ab4GNb2Ma6)Mb4GNb4GN	GP_{21}	G00251
**19**	FA1	GNb2Ma3(Ma6)Mb4GNb4(Fa6)GN	GP_1	G10770
**20**	FA1G1	Ab4GNb2Ma3(Ma6)Mb4GNb4(Fa6)GN	–	–
**21**	FA1G1S1	NNa3Ab4GNb2Ma3(Ma6)Mb4GNb4(Fa6)GN	GP_{15}	–
**22**	FA2	GNb2Ma3(GNb2Ma6)Mb4GNb4(Fa6)GN	GP_4, GP_5	G00016
**23**	FA2[3]BG1	Ab4GNb2Ma3(GNb4)(GNb2Ma6)Mb4GNb4(Fa6)GN	GP_{11}, GP_{12}	–
**24**	FA2[3]BG1S1	NNa3Ab4GNb2Ma3(GNb4)(GNb2Ma6)Mb4GNb4(Fa6)GN	GP_{17}	–
**25**	FA2[3]BG2S1	NNa3Ab4GNb2Ma3(GNb4)(Ab4GNb2Ma6)Mb4GNb4(Fa6)GN	GP_{19}	–
**26**	FA2[3]G1	Ab4GNb2Ma3(GNb2Ma6)Mb4GNb4(Fa6)GN	GP_9	–

**27**	FA2[3]G1S1	NNa3Ab4GNb2Ma3(GNb2Ma6)Mb4GNb4(Fa6)GN	GP_{16}	–
**28**	FA2[3]G2S1	NNa3Ab4GNb2Ma3(Ab4GNb2Ma6)Mb4GNb4(Fa6)GN	GP_{18}	–
**29**	FA2[6]BG1	GNb2Ma3(GNb4)(Ab4GNb2Ma6)Mb4GNb4(Fa6)GN	GP_{10}	–
**30**	FA2[6]BG1S1	GNb2Ma3(GNb4)(NNa3Ab4GNb2Ma6)Mb4GNb4(Fa6)GN	GP_{16}	–
**31**	FA2[6]BG2S1	Ab4GNb2Ma3(GNb4)(NNa3Ab4GNb2Ma6)Mb4GNb4(Fa6)GN	GP_{19}	–
**32**	FA2[6]G1	GNb2Ma3(Ab4GNb2Ma6)Mb4GNb4(Fa6)GN	GP_{8}	–
**33**	FA2[6]G1S1	GNb2Ma3(NNa3Ab4GNb2Ma6)Mb4GNb4(Fa6)GN	GP_{16}	–
**34**	FA2[6]G2S1	Ab4GNb2Ma3(NNa3Ab4GNb2Ma6)Mb4GNb4(Fa6)GN	GP_{18}	–
**35**	FA2B	GNb2Ma3(GNb4)(GNb2Ma6)Mb4GNb4(Fa6)GN	GP_{6}, GP_{7}	–
**36**	FA2BG2	Ab4GNb2Ma3(GNb4)(Ab4GNb2Ma6)Mb4GNb4(Fa6)GN	GP_{15}	–
**37**	FA2BG2S2	NNa3Ab4GNb2Ma3(GNb4)(NNa3Ab4GNb2Ma6)Mb4GNb4(Fa6)GN	GP_{24}	–
**38**	FA2G2	Ab4GNb2Ma3(Ab4GNb2Ma6)Mb4GNb4(Fa6)GN	GP_{13}, GP_{14}, GP_{15}	G00017
**39**	FA2G2S2	NNa3Ab4GNb2Ma3(NNa3Ab4GNb2Ma6)Mb4GNb4(Fa6)GN	GP_{23}	G00018
**40**	M3A1	GNb2Ma3(Ma6)Mb4GNb4GN	–	G00014
**41**	M4A1	GNb2Ma3(Ma6Ma6)Mb4GNb4GN	–	–
**42**	M4A1G1	Ab4GNb2Ma3(Ma6Ma6)Mb4GNb4GN	–	–
**43**	M4A1G1S1	NNa3Ab4GNb2Ma3(Ma6Ma6)Mb4GNb4GN	GP_{16}	–
**44**	M5	Ma3(Ma3(Ma6)Ma6)Mb4GNb4GN	GP_{5}	G00012
**45**	M5A1	GNb2Ma3(Ma3(Ma6)Ma6)Mb4GNb4GN	–	G00013

Table S2. List of enzymes of the IgG N-glycosylation model

Enzymes	EC numbers	Names [29]	Distribution of enzymes across the four Golgi compartments[1]			
			I	II	III	IV
GnT I	2.4.1.101	α-1,3-mannosyl-glycoprotein 2-β-N-acetylglucosaminyltransferase	0.30	0.30	0.30	0.10
GnT II	2.4.1.143	α-1,6-mannosyl-glycoprotein 2-β-N-acetylglucosaminyltransferase	0.15	0.45	0.30	0.10
GnT III	2.4.1.144	β-1,4-mannosyl-glycoprotein 4-β-N-acetylglucosaminyltransferase	0.15	0.45	0.30	0.10
Man II	3.2.1.114	Mannosyl-oligosaccharide 1,3-1,6-α-mannosidase	0.15	0.45	0.30	0.10
FucT	2.4.1.68	Glycoprotein 6-α-L-fucosyltransferase	0.00	0.65	0.25	0.10
GalT	2.4.1.38	β-N-acetylglucosaminylglycopeptide β-1,4-galactosyltransferase	0.00	0.05	0.20	0.75
SiaT	2.4.3.1 (ex-2.4.99.1) 2.4.3.6 (ex-2.4.99.6)	β-galactoside α-2,6-sialyltransferase<br>N-acetyllactosaminide α-2,3-sialyltransferase	0.00	0.05	0.20	0.75

[1] Values for GnT II, GnT III, Man II, GalT, and SiaT were adopted from the model by Krambeck et al. [30], whereas values for GnT I and FucT were estimated using experimental data from Pučić et al. [27].

Table S3. List of reactions of the IgG N-glycosylation model

№	Reactions	Enzymes	Parameters		KEGG ID[2]
			kf	*Km*	
**J01**	M5 → M5A1	GnT I	*kfGnT1*	*KmGnT1*	R05983
**J02**	M5A1 → M4A1	Man II	*kfMan*	*KmMan2_5*	R05984*
**J03**	M4A1 → M3A1	Man II	*kfMan*	*KmMan2_6*	R05984*
**J04**	M4A1 → M4A1G1	GalT	*k04_11_12 · kfGalT*	*KmGalT*	R04495
**J05**	M3A1 → FA1	FucT	*kfFucT*	*KmFucT*	R09319
**J06**	M3A1 → A2	GnT II	*kfGnT2*	*KmGnT2*	R05985, R04653
**J07**	M4A1G1 → M4A1G1S1	SiaT	*k07_21_22 · kfSiaT*	*KmSiaT*	R04596, R05580, R06188
**J08**	FA1 → FA2	GnT II	*kfGnT2*	*KmGnT2*	R04653
**J09**	A2 → FA2	FucT	*kfFucT*	*KmFucT*	R05988
**J10**	FA1 → FA1G1	GalT	*k10_15_16 · kfGalT*	*KmGalT*	R04495
**J11**	A2 → A2[6]G1	GalT	*k04_11_12 · kfGalT*	*KmGalT*	R04495
**J12**	A2 → A2[3]G1	GalT	*k04_11_12 · kfGalT*	*KmGalT*	R04495
**J13**	A2 → A2B	GnT III	*kfGnT3*	*KmGnT3*	R05986, R07259
**J14**	FA2 → FA2B	GnT III	*kfGnT3*	*KmGnT3*	R07259
**J15**	FA2 → FA2[6]G1	GalT	*k10_15_16 · kfGalT*	*KmGalT*	R05989*, R04495
**J16**	FA2 → FA2[3]G1	GalT	*k10_15_16 · kfGalT*	*KmGalT*	R05989*, R04495
**J17**	FA1G1 → FA1G1S1	SiaT	*k17_26_29 · kfSiaT*	*KmSiaT*	R04596, R05580, R06188
**J18**	A2[6]G1 → A2G2	GalT	*k18_19_31_33 · kfGalT*	*KmGalT*	R04495
**J19**	A2[3]G1 → A2G2	GalT	*k18_19_31_33 · kfGalT*	*KmGalT*	R04495
**J20**	A2[3]G1 → A2[3]G1S1	SiaT	*k07_21_22 · kfSiaT*	*KmSiaT*	R04596, R05580, R06188
**J21**	A2[6]G1 → A2[6]G1S1	SiaT	*k07_21_22 · kfSiaT*	*KmSiaT*	R04596, R05580, R06188
**J22**	A2B → A2[6]BG1	GalT	*k22_23 · kfGalT*	*KmGalT*	R04495
**J23**	A2B → A2[3]BG1	GalT	*k22_23 · kfGalT*	*KmGalT*	R04495
**J24**	FA2B → FA2[3]BG1	GalT	*k24_25 · kfGalT*	*KmGalT*	R04495

[2] References marked with an asterisk correspond to general reactions that include specific reactions Jxx as their components.

**J25**	FA2B → FA2[6]BG1	GalT	*k24_25 · kfGalT*	*KmGalT*	R04495
**J26**	FA2[6]G1 → FA2[6]G1S1	SiaT	*k17_26_29 · kfSiaT*	*KmSiaT*	R04596, R05580, R06188
**J27**	FA2[6]G1 → FA2G2	GalT	*k27_42 · kfGalT*	*KmGalT*	R05989*, R04495
**J28**	FA2[3]G1 → FA2G2	GalT	*k28_45 · kfGalT*	*KmGalT*	R05989*, R04495
**J29**	FA2[3]G1 → FA2[3]G1S1	SiaT	*k17_26_29 · kfSiaT*	*KmSiaT*	R04596, R05580, R06188
**J30**	A2G2 → A2[3]G2S1	SiaT	*k30_47 · kfSiaT*	*KmSiaT*	R04596, R05580, R06188
**J31**	A2[3]G1S1 → A2[3]G2S1	GalT	*k18_19_31_33 · kfGalT*	*KmGalT*	R04495
**J32**	A2G2 → A2[6]G2S1	SiaT	*k32_46 · kfSiaT*	*KmSiaT*	R04596, R05580, R06188
**J33**	A2[6]G1S1 → A2[6]G2S1	GalT	*k18_19_31_33 · kfGalT*	*KmGalT*	R04495
**J34**	A2[6]BG1 → A2[6]BG1S1	SiaT	*k34_37 · kfSiaT*	*KmSiaT*	R04596, R05580, R06188
**J35**	A2[6]BG1 → A2BG2	GalT	*k35_36_48_51 · kfGalT*	*KmGalT*	R04495
**J36**	A2[3]BG1 → A2BG2	GalT	*k35_36_48_51 · kfGalT*	*KmGalT*	R04495
**J37**	A2[3]BG1 → A2[3]BG1S1	SiaT	*k34_37 · kfSiaT*	*KmSiaT*	R04596, R05580, R06188
**J38**	FA2[3]BG1 → FA2BG2	GalT	*k38_53 · kfGalT*	*KmGalT*	R04495
**J39**	FA2[6]BG1 → FA2BG2	GalT	*k39_55 · kfGalT*	*KmGalT*	R04495
**J40**	FA2[3]BG1 → FA2[3]BG1S1	SiaT	*k40_41 · kfSiaT*	*KmSiaT*	R04596, R05580, R06188
**J41**	FA2[6]BG1 → FA2[6]BG1S1	SiaT	*k40_41 · kfSiaT*	*KmSiaT*	R04596, R05580, R06188
**J42**	FA2[6]G1S1 → FA2[6]G2S1	GalT	*k27_42 · kfGalT*	*KmGalT*	R04495
**J43**	FA2G2 → FA2[6]G2S1	SiaT	*k43_57 · kfSiaT*	*KmSiaT*	R05990*, R04596, R05580, R06188
**J44**	FA2G2 → FA2[3]G2S1	SiaT	*k44_56 · kfSiaT*	*KmSiaT*	R05990*, R04596, R05580, R06188
**J45**	FA2[3]G1S1 → FA2[3]G2S1	GalT	*k28_45 · kfGalT*	*KmGalT*	R04495
**J46**	A2[3]G2S1 → A2G2S2	SiaT	*k32_46 · kfSiaT*	*KmSiaT*	R05905, R04596, R05580, R06188
**J47**	A2[6]G2S1 → A2G2S2	SiaT	*k30_47 · kfSiaT*	*KmSiaT*	R05906, R04596, R05580, R06188
**J48**	A2[6]BG1S1 → A2[6]BG2S1	GalT	*k35_36_48_51 · kfGalT*	*KmGalT*	R04495
**J49**	A2BG2 → A2[6]BG2S1	SiaT	*k49_59 · kfSiaT*	*KmSiaT*	R04596, R05580, R06188
**J50**	A2BG2 → A2[3]BG2S1	SiaT	*k50_58 · kfSiaT*	*KmSiaT*	R04596, R05580, R06188
**J51**	A2[3]BG1S1 → A2[3]BG2S1	GalT	*k35_36_48_51 · kfGalT*	*KmGalT*	R04495
**J52**	FA2BG2 → FA2[3]BG2S1	SiaT	*k52_61 · kfSiaT*	*KmSiaT*	R04596, R05580, R06188

**J53**	FA2[3]BG1S1 → FA2[3]BG2S1	GalT	*k38_53 · kfGalT*	*KmGalT*	R04495
**J54**	FA2BG2 → FA2[6]BG2S1	SiaT	*k54_60 · kfSiaT*	*KmSiaT*	R04596, R05580, R06188
**J55**	FA2[6]BG1S1 → FA2[6]BG2S1	GalT	*k39_55 · kfGalT*	*KmGalT*	R04495
**J56**	FA2[6]G2S1 → FA2G2S2	SiaT	*k44_56 · kfSiaT*	*KmSiaT*	R05990*, R04596, R05580, R06188
**J57**	FA2[3]G2S1 → FA2G2S2	SiaT	*k43_57 · kfSiaT*	*KmSiaT*	R05990*, R04596, R05580, R06188
**J58**	A2[6]BG2S1 → A2BG2S2	SiaT	*k50_58 · kfSiaT*	*KmSiaT*	R04596, R05580, R06188
**J59**	A2[3]BG2S1 → A2BG2S2	SiaT	*k49_59 · kfSiaT*	*KmSiaT*	R04596, R05580, R06188
**J60**	FA2[3]BG2S1 → FA2BG2S2	SiaT	*k54_60 · kfSiaT*	*KmSiaT*	R04596, R05580, R06188
**J61**	FA2[6]BG2S1 → FA2BG2S2	SiaT	*k52_61 · kfSiaT*	*KmSiaT*	R04596, R05580, R06188

Table S4. List of parameters of the IgG N-glycosylation model

№	Parameters	Initial values	Units	Source	№	Parameters	Initial values	Units	Source
01	*kfFucT*	253.0	min^{-1}	Bennun et al., 2013 [31]	20	*k18_19_31_33*	2.59	−	Fitted
02	*kfGalT*	8712.0	min^{-1}	Bennun et al., 2013 [31]	21	*k22_23*	12.14	−	Fitted
03	*kfGnT1*	990.0	min^{-1}	Bennun et al., 2013 [31]	22	*k24_25*	0.61	−	Fitted
04	*kfGnT2*	1320.0	min^{-1}	Bennun et al., 2013 [31]	23	*k27_42*	0.47	−	Fitted
05	*kfGnT3*	607.2	min^{-1}	Bennun et al., 2013 [31]	24	*k28_45*	0.94	−	Fitted
06	*kfMan*	1923.75	min^{-1}	Bennun et al., 2013 [31]	25	*k30_47*	2.75	−	Fitted
07	*kfSiaT*	484.1	min^{-1}	Bennun et al., 2013 [31]	26	*k32_46*	9.1	−	Fitted
08	*KmFucT*	25.0	μM	Bennun et al., 2013 [31]	27	*k34_37*	0.43	−	Fitted
09	*KmGalT*	150.0	μM	Bennun et al., 2013 [31]	28	*k35_36_48_51*	0.09	−	Fitted
10	*KmGnT1*	260.0	μM	Bennun et al., 2013 [31]	29	*k38_53*	3.56	−	Fitted
11	*KmGnT2*	190.0	μM	Bennun et al., 2013 [31]	30	*k39_55*	0.13	−	Fitted
12	*KmGnT3*	190.0	μM	Bennun et al., 2013 [31]	31	*k40_41*	0.31	−	Fitted
13	*KmMan2_5*	200.0	μM	Bennun et al., 2013 [31]	32	*k43_57*	0.53	−	Fitted
14	*KmMan2_6*	100.0	μM	Bennun et al., 2013 [31]	33	*k44_56*	1.88	−	Fitted
15	*KmSiaT*	260.0	μM	Bennun et al., 2013 [31]	34	*k49_59*	4.04	−	Fitted
16	*k04_11_12*	1.3	−	Fitted	35	*k50_58*	2.47	−	Fitted
17	*k07_21_22*	1.65	−	Fitted	36	*k52_61*	9.06	−	Fitted
18	*k10_15_16*	0.5	−	Fitted	37	*k54_60*	2.57	−	Fitted
19	*k17_26_29*	0.38	−	Fitted					Fitted

Table S5. Equations for calculating chromatographic GP peaks

Glycan peaks	Equations [27]
GP_1	FA1 / GP_{peaks} · 100.0
GP_2	A2 / GP_{peaks} · 100.0
GP_4	0.995 · FA2 / GP_{peaks} · 100.0
GP_5	(M5 + 0.005 · FA2) / GP_{peaks} · 100.0
GP_6	(0.969 · FA2B + A2[6]G1) / GP_{peaks} · 100.0
GP_7	(A2[3]G1 + 0.031 · FA2B / GP_{peaks} · 100.0
GP_8	(FA2[6]G1 + A2[3]BG1 + A2[6]BG1) / GP_{peaks} · 100.0
GP_9	FA2[3]G1 / GP_{peaks} · 100.0
GP_{10}	FA2[6]BG1 / GP_{peaks} · 100.0
GP_{11}	0.896 · FA2[3]BG1 / GP_{peaks} · 100.0
GP_{12}	(A2G2 + 0.104 · FA2[3]BG1) / GP_{peaks} · 100.0
GP_{13}	(A2BG2 + 0.003 · FA2G2) / GP_{peaks} · 100.0
GP_{14}	0.992 · FA2G2 / GP_{peaks} · 100.0
GP_{15}	(FA2BG2 + FA1G1S1 + A2[3]G1S1 + A2[6]G1S1 + 0.005 · FA2G2) / GP_{peaks} · 100.0
GP_{16}	(FA2[6]G1S1 + M4A1G1S1 + A2[3]BG1S1 + A2[6]BG1S1 + FA2[3]G1S1 + FA2[6]BG1S1) / GP_{peaks} · 100.0
GP_{17}	(A2[3]G2S1 + A2[6]G2S1 + FA2[3]BG1S1) / GP_{peaks} · 100.0
GP_{18}	(A2[3]BG2S1 + A2[6]BG2S1 + FA2[3]G2S1 + FA2[6]G2S1) / GP_{peaks} · 100.0
GP_{19}	(FA2[6]BG2S1 + FA2[3]BG2S1) / GP_{peaks} · 100.0
GP_{21}	A2G2S2 / GP_{peaks} · 100.0
GP_{22}	A2BG2S2 / GP_{peaks} · 100.0
GP_{23}	FA2G2S2 / GP_{peaks} · 100.0
GP_{24}	FA2BG2S2 / GP_{peaks} · 100.0
GP_{peaks}	FA1 + A2 + FA2 + M5 + FA2B + A2[6]G1 + A2[3]G1 + FA2[6]G1 + A2[3]BG1 + A2[6]BG1 + FA2[3]G1 + FA2[6]BG1 + FA2[3]BG1 + A2G2 + A2BG2 + FA2G2 + FA2BG2 + FA1G1S1 + A2[3]G1S1 + A2[6]G1S1 + FA2[6]G1S1 + M4A1G1S1 + A2[3]BG1S1 + A2[6]BG1S1 + FA2[3]G1S1 + FA2[6]BG1S1 + A2[3]G2S1 + A2[6]G2S1 + FA2[3]BG1S1 + FA2[3]G2S1 + FA2[6]G2S1 + A2[3]BG2S1 + A2[6]BG2S1 + FA2[6]BG2S1 + FA2[3]BG2S1 + A2G2S2 + A2BG2S2 + FA2G2S2 + FA2BG2S2

Table S6. Comparison of experimental [27] and simulated glycan peak values in the Korčula (n = 915) and Vis (n = 890) populations

Glycan peaks	Korčula			Vis		
	Percentage experimental values, mean ± SD	Percentage simulated values, mean ± SD	*P*-values, Mann-Whitney test	Percentage experimental values, mean ± SD	Percentage simulated values, mean ± SD	*P*-values, Mann-Whitney test
GP_1	0.18 ± 0.12	0.19 ± 0.13	0.4133	0.20 ± 0.15	0.20 ± 0.14	0.0002
GP_2	0.86 ± 0.61	0.84 ± 0.47	0.5373	0.86 ± 0.50	0.87 ± 0.40	0.5831
GP_4	20.38 ± 5.98	20.94 ± 5.02	0.0160	20.80 ± 6.11	21.21 ± 5.01	0.3148
GP_5	0.31 ± 0.12	0.32 ± 0.13	0.5159	0.32 ± 0.10	0.32 ± 0.11	0.0002
GP_6	5.58 ± 1.60	5.43 ± 1.52	0.1343	5.42 ± 1.59	5.52 ± 1.52	0.4705
GP_7	0.77 ± 0.43	0.70 ± 0.25	0.2988	0.75 ± 0.34	0.73 ± 0.22	0.0660
GP_8	16.09 ± 1.88	16.18 ± 0.80	0.5857	16.31 ± 1.82	16.31 ± 0.75	0.0202
GP_9	7.98 ± 1.26	8.11 ± 0.90	0.0008	7.88 ± 1.09	8.14 ± 0.80	1.3090e-08
GP_{10}	4.67 ± 0.93	4.72 ± 0.68	0.0044	4.73 ± 0.89	4.72 ± 0.61	0.1714
GP_{11}	0.79 ± 0.17	0.77 ± 0.20	0.4916	0.77 ± 0.16	0.78 ± 0.19	0.2762
GP_{12}	1.12 ± 0.58	1.07 ± 0.41	0.6520	1.06 ± 0.50	1.14 ± 0.38	0.0464
GP_{13}	0.23 ± 0.07	0.26 ± 0.09	6.2609e-19	0.28 ± 0.09	0.27 ± 0.09	2.8546e-07
GP_{14}	11.23 ± 3.46	11.81 ± 3.66	0.0018	11.43 ± 3.51	11.86 ± 3.67	0.0819
GP_{15}	1.54 ± 0.34	1.54 ± 0.25	0.3470	1.45 ± 0.34	1.57 ± 0.25	2.1186e-14
GP_{16}	3.23 ± 0.46	3.35 ± 0.58	1.3885e-06	3.19 ± 0.47	3.24 ± 0.55	4.2748e-11
GP_{17}	2.92 ± 1.58	2.59 ± 1.03	0.0084	3.03 ± 1.36	2.66 ± 0.85	1.7097e-14
GP_{18}	9.31 ± 2.64	9.28 ± 2.31	0.4934	8.91 ± 2.53	8.98 ± 2.26	0.0001
GP_{19}	2.48 ± 0.50	2.44 ± 0.52	0.3350	2.51 ± 0.53	2.45 ± 0.51	0.0866
GP_{21}	4.13 ± 2.80	3.81 ± 1.83	0.3518	3.85 ± 1.84	3.68 ± 1.27	0.3299
GP_{22}	0.33 ± 0.12	0.35 ± 0.12	1.1370e-06	0.39 ± 0.12	0.34 ± 0.10	1.1873e-12
GP_{23}	2.32 ± 0.63	2.43 ± 0.73	0.0039	2.03 ± 0.64	2.24 ± 0.67	2.5899e-34
GP_{24}	2.80 ± 0.63	2.87 ± 0.55	0.0008	2.76 ± 0.63	2.76 ± 0.53	9.3734e-07

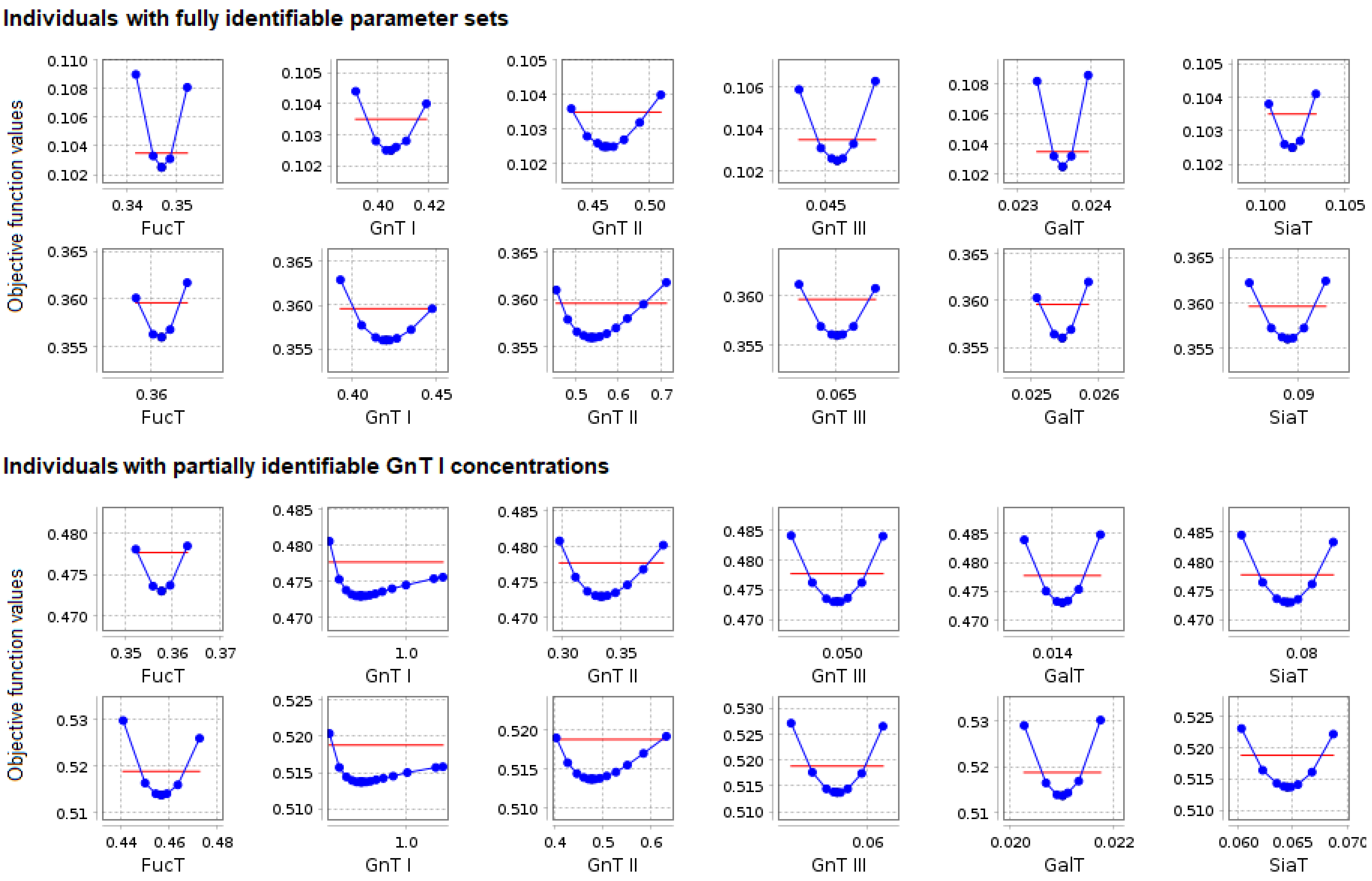

Figure S1. Results of the parameter identifiability analysis for the IgG N-glycosylation model. The graphs show the objective function value (*Y*-axis) plotted against total enzyme concentration fixed during optimization (*X*-axis). Parabolic curves indicate that deviations from the optimized parameter value (parabola vertex) increase the objective function, reflecting a poorer fit to the experimental data. The red lines mark a 1% increase threshold in the objective function, which serves as the termination criterion for the analysis. Results are presented for four individuals: two with fully identifiable parameters and two with partially identifiable GnT I levels. Each row corresponds to one subject, and each column represents one of the six model parameters.

Table S7. Median total enzyme concentrations estimated for the Korčula population (*n* = 915), with ±50% ranges applied in the global Sobol sensitivity analysis.

Enzymes	Median values (µM)	Ranges (µM)
FucT	0.4260297	[0.21301485, 0.63904455]
GalT	0.02270569	[0.011352845, 0.034058535]
GnT I	0.5331037	[0.26655185, 0.79965555]
GnT II	0.5881121	[0.29405605, 0.88216815]
GnT III	0.05578655	[0.027893275, 0.083679825]
SiaT	0.08896045	[0.044480225, 0.133440675]

Table S8. First-order and total-effect Sobol global sensitivity indices quantifying the contributions of six total enzyme concentrations to the variance observed in twenty-two glycan peaks in the Korčula population (n = 915).

Glycan peaks	First-order sensitivity indices						Total-effect sensitivity indices					
	FucT	GnT I	GnT II	GnT III	GalT	SiaT	FucT	GnT I	GnT II	GnT III	GalT	SiaT
GP_1	0.174	0.067	0.726	0.001	0.007	0.000	0.256	0.110	0.773	0.001	0.025	0.000
GP_2	0.618	0.000	0.000	0.010	0.213	0.000	0.825	0.001	0.000	0.019	0.424	0.000
GP_4	0.195	0.003	0.000	0.039	0.695	0.000	0.224	0.002	0.000	0.052	0.751	0.000
GP_5	0.001	0.940	0.000	0.000	0.003	0.000	0.002	0.974	0.000	0.000	0.005	0.000
GP_6	0.036	0.001	0.000	0.213	0.647	0.000	0.060	0.000	0.000	0.262	0.722	0.001
GP_7	0.756	0.000	0.000	0.002	0.184	0.007	0.845	0.001	0.000	0.011	0.281	0.012
GP_8	0.051	0.061	0.000	0.097	0.303	0.149	0.355	0.040	0.010	0.322	0.426	0.183
GP_9	0.550	0.003	0.000	0.115	0.243	0.008	0.607	0.003	0.000	0.135	0.269	0.014
GP_{10}	0.228	0.000	0.000	0.654	0.095	0.006	0.271	0.001	0.000	0.662	0.092	0.011
GP_{11}	0.197	0.001	0.000	0.325	0.384	0.000	0.228	0.000	0.000	0.360	0.454	0.001
GP_{12}	0.587	0.000	0.000	0.005	0.096	0.128	0.766	0.000	0.000	0.015	0.179	0.234
GP_{13}	0.268	0.001	0.001	0.065	0.393	0.130	0.378	0.001	0.001	0.113	0.496	0.200
GP_{14}	0.029	0.000	0.000	0.028	0.791	0.099	0.049	0.000	0.000	0.037	0.823	0.129
GP_{15}	0.011	0.000	0.001	0.192	0.224	0.197	0.221	0.001	0.001	0.277	0.509	0.358
GP_{16}	0.004	0.001	0.000	0.040	0.348	0.553	0.013	0.001	0.000	0.046	0.392	0.577
GP_{17}	0.805	0.001	0.000	0.010	0.089	0.025	0.873	0.000	0.000	0.016	0.141	0.039
GP_{18}	0.034	0.000	0.000	0.026	0.809	0.119	0.056	0.000	0.000	0.033	0.827	0.134
GP_{19}	0.071	0.001	0.000	0.341	0.506	0.045	0.101	0.001	0.000	0.382	0.522	0.058
GP_{21}	0.833	0.000	0.000	0.010	0.036	0.057	0.875	0.000	0.000	0.015	0.058	0.118
GP_{22}	0.398	0.001	0.002	0.127	0.187	0.156	0.485	0.001	0.002	0.181	0.257	0.242
GP_{23}	0.038	0.001	0.000	0.016	0.184	0.713	0.056	0.001	0.000	0.025	0.233	0.755
GP_{24}	0.090	0.002	0.000	0.393	0.225	0.296	0.116	0.003	0.000	0.422	0.244	0.326

Table S9. Local sensitivities of glycan peaks to median total enzyme concentrations (estimated in the Korčula population, *n* = 915), as well as to concentrations in the second (II), third (III), and fourth (IV) Golgi compartments for FucT, GalT, and SiaT.

Glycan peaks	FucT concentration				GalT concentration				SiaT concentration			
	Total	II	III	IV	Total	II	III	IV	Total	II	III	IV
GP_1	1.3644	0.5162	0.3400	0.5082	-0.2163	0.0125	0.0032	-0.2320	-0.0021	-0.0001	-0.0004	-0.0016
GP_2	-4.1686	-2.4028	-1.2611	-0.5048	-1.3682	-0.1565	-0.3527	-0.8590	-0.0021	-0.0001	-0.0004	-0.0016
GP_4	0.6537	0.4366	0.1755	0.0416	-1.3892	-0.1056	-0.3360	-0.9476	-0.0021	-0.0001	-0.0004	-0.0016
GP_5	0.2410	0.1611	0.0648	0.0151	-0.5104	-0.0383	-0.1234	-0.3487	-0.0021	-0.0001	-0.0004	-0.0016
GP_6	0.3748	0.3707	0.0168	-0.0127	-1.4543	-0.0797	-0.3471	-1.0276	-0.0223	-0.0016	-0.0075	-0.0132
GP_7	-2.6685	-1.7075	-0.7673	-0.1936	-0.9315	0.0109	-0.1590	-0.7833	-0.1660	-0.0125	-0.0579	-0.0957
GP_8	-0.0565	-0.1236	0.0509	0.0163	-0.1098	-0.0150	0.0083	-0.1031	-0.1335	-0.0050	-0.0304	-0.0981
GP_9	0.5514	0.3780	0.1460	0.0275	-0.4479	-0.0257	-0.0529	-0.3693	-0.0780	-0.0014	-0.0149	-0.0617
GP_{10}	0.5377	0.4828	0.0545	0.0003	0.2530	0.0088	0.0627	0.1815	-0.1131	-0.0012	-0.0163	-0.0956
GP_{11}	0.7618	0.6385	0.1129	0.0104	-1.1823	-0.0614	-0.2508	-0.8701	-0.0278	-0.0005	-0.0061	-0.0212
GP_{12}	-3.5887	-2.3955	-0.9786	-0.2146	1.4325	0.1608	0.3206	0.9510	-1.1669	-0.0246	-0.1790	-0.9633
GP_{13}	-1.5312	-1.3195	-0.1965	-0.0152	2.0483	0.0680	0.3510	1.6293	-1.0210	-0.0164	-0.1176	-0.8871
GP_{14}	0.1968	0.1307	0.0566	0.0095	1.8416	0.0726	0.3260	1.4431	-0.6453	-0.0034	-0.0489	-0.5931
GP_{15}	-0.1500	-0.0306	-0.0996	-0.0198	0.8747	0.0534	0.0810	0.7403	-0.6997	0.0081	-0.0028	-0.7050
GP_{16}	0.1024	0.0165	0.0710	0.0148	-0.9503	-0.0067	-0.1286	-0.8151	1.0456	0.0313	0.2170	0.7973
GP_{17}	-3.7510	-2.5986	-0.9753	-0.1771	1.2486	0.2335	0.3801	0.6350	-0.5316	-0.0079	-0.0888	-0.4349
GP_{18}	0.2144	0.1409	0.0632	0.0103	1.2554	0.0555	0.2607	0.9392	0.3897	0.0023	0.0316	0.3557
GP_{19}	0.3994	0.3854	0.0199	-0.0059	1.1411	0.0466	0.2141	0.8804	-0.4069	-0.0017	-0.0395	-0.3657
GP_{21}	-3.5963	-2.5385	-0.9126	-0.1452	0.7266	0.2494	0.3131	0.1640	0.7029	0.0124	0.1130	0.5774
GP_{22}	-1.6436	-1.4323	-0.1985	-0.0129	1.0363	0.0418	0.2090	0.7854	0.9527	0.0101	0.0963	0.8463
GP_{23}	0.3502	0.2518	0.0853	0.0130	0.6684	0.0312	0.1641	0.4731	1.5865	0.0069	0.1102	1.4694
GP_{24}	0.4660	0.4383	0.0313	-0.0035	0.6205	0.0282	0.1620	0.4302	0.7746	0.0028	0.0606	0.7112

Table S10. Local sensitivities of glycan peaks to median total enzyme concentrations (estimated in the Korčula population, *n* = 915), as well as to concentrations in the first (I), second (II), third (III), and fourth (IV) Golgi compartments for GnT I, GnT II, and GnT III.

Glycan peaks	GnT I concentration					GnT II concentration					GnT III concentration				
	Total	I	II	III	IV	Total	I	II	III	IV	Total	I	II	III	IV
GP_1	-0.7039	-0.4417	-0.2504	-0.0388	0.0271	-2.0223	-0.0200	-0.2491	-0.6857	-1.0674	0.0552	0.0219	0.0247	0.0078	0.0009
GP_2	-0.1288	-0.1022	-0.0496	0.0146	0.0083	0.0426	-0.0155	-0.0260	0.0175	0.0665	-0.3310	-0.1158	-0.1456	-0.0568	-0.0128
GP_4	0.0344	0.0174	0.0112	0.0042	0.0016	-0.0078	-0.0031	-0.0048	-0.0022	0.0023	-0.3287	-0.0242	-0.1547	-0.1148	-0.0350
GP_5	-2.4403	-0.8614	-0.6058	-0.5510	-0.4221	-0.0061	-0.0012	-0.0024	-0.0020	-0.0005	-0.1215	-0.0089	-0.0572	-0.0424	-0.0129
GP_6	0.0027	-0.0019	0.0000	0.0030	0.0015	-0.0055	-0.0017	-0.0078	0.0013	0.0027	0.8064	-0.0052	0.3705	0.3289	0.1122
GP_7	-0.0126	-0.0213	-0.0072	0.0120	0.0039	0.0481	-0.0083	0.0049	0.0259	0.0257	-0.0121	-0.0823	-0.0048	0.0510	0.0239
GP_8	0.0399	0.0253	0.0119	0.0017	0.0010	0.0230	0.0110	0.0114	0.0006	-0.0001	0.0456	0.0897	0.0162	-0.0453	-0.0150
GP_9	0.0201	0.0101	0.0066	0.0022	0.0012	-0.0078	-0.0028	-0.0038	-0.0019	0.0007	-0.3060	-0.0220	-0.1514	-0.1071	-0.0255
GP_{10}	-0.0273	-0.0167	-0.0094	-0.0018	0.0006	-0.0173	-0.0007	-0.0106	-0.0037	-0.0023	0.9407	0.0068	0.4710	0.3737	0.0893
GP_{11}	-0.0033	-0.0038	-0.0017	0.0010	0.0011	-0.0148	-0.0008	-0.0105	-0.0027	-0.0008	0.9278	0.0068	0.4494	0.3706	0.1010
GP_{12}	-0.0215	-0.0261	-0.0100	0.0111	0.0035	0.0648	-0.0084	0.0151	0.0330	0.0251	-0.1760	-0.0945	-0.0785	-0.0065	0.0035
GP_{13}	0.0688	0.0505	0.0190	-0.0010	0.0003	0.0881	0.0398	0.0443	0.0064	-0.0024	0.7657	0.3220	0.3614	0.0779	0.0044
GP_{14}	-0.0076	-0.0045	-0.0023	-0.0013	0.0004	-0.0073	-0.0025	-0.0018	-0.0017	-0.0013	-0.2954	-0.0219	-0.1480	-0.1027	-0.0227
GP_{15}	-0.0229	-0.0159	-0.0082	0.0002	0.0011	-0.0277	-0.0023	-0.0103	-0.0067	-0.0084	0.6812	-0.0124	0.3370	0.2839	0.0727
GP_{16}	-0.0186	-0.0114	-0.0076	-0.0023	0.0028	0.0141	0.0106	0.0063	-0.0016	-0.0012	0.2536	0.0918	0.1226	0.0353	0.0039
GP_{17}	0.0013	-0.0110	-0.0018	0.0112	0.0029	0.0739	-0.0083	0.0259	0.0362	0.0201	-0.2674	-0.1061	-0.1234	-0.0342	-0.0036
GP_{18}	-0.0111	-0.0057	-0.0034	-0.0021	0.0002	-0.0080	-0.0014	-0.0016	-0.0028	-0.0022	-0.2381	-0.0118	-0.1212	-0.0867	-0.0184
GP_{19}	-0.0375	-0.0221	-0.0127	-0.0030	0.0003	-0.0171	-0.0006	-0.0100	-0.0036	-0.0029	0.9433	0.0072	0.4763	0.3738	0.0859
GP_{21}	0.0076	-0.0054	0.0009	0.0098	0.0023	0.0712	-0.0087	0.0296	0.0349	0.0155	-0.2679	-0.1138	-0.1242	-0.0282	-0.0017
GP_{22}	0.0658	0.0511	0.0176	-0.0028	-0.0002	0.0931	0.0457	0.0474	0.0041	-0.0041	0.9588	0.3730	0.4595	0.1161	0.0102
GP_{23}	-0.0220	-0.0121	-0.0067	-0.0032	0.0000	-0.0163	-0.0038	-0.0050	-0.0044	-0.0032	-0.2631	-0.0299	-0.1330	-0.0837	-0.0166
GP_{24}	-0.0481	-0.0278	-0.0161	-0.0042	0.0001	-0.0230	-0.0013	-0.0123	-0.0055	-0.0038	0.9588	0.0028	0.4927	0.3815	0.0818

Table S11. Results of linear regression analysis predicting age from enzyme levels in the Korčula (*n* = 915) and Vis (*n* = 890) populations, with model performance assessed by leave-one-subject-out cross-validation (LOOCV)

Population	Enzymes	*P*-values from linear regression models	Bonferroni corrections	Benjamini-Hochberg corrections	Mean squared errors of LOOCV
Korčula (*n* = 915)	FucT	3.684276e-06	2.210566e-05	5.526415e-06	197.5633
	GnT I	1.982993e-03	1.189796e-02	1.982993e-03	200.1228
	GnT II	4.062769e-11	2.437661e-10	8.125538e-11	192.7427
	GnT III	6.592983e-19	3.955790e-18	1.977895e-18	185.5010
	GalT	5.085576e-93	3.051346e-92	3.051346e-92	127.8989
	SiaT	1.245156e-04	7.470934e-04	1.494187e-04	198.9261
Vis (*n* = 890)	FucT	5.870461e-13	3.522277e-12	1.174092e-12	234.5343
	GnT I	4.570660e-03	2.742396e-02	4.570660e-03	246.2971
	GnT II	7.629581e-08	4.577749e-07	1.144437e-07	240.6342
	GnT III	4.663502e-13	2.798101e-12	1.174092e-12	234.4322
	GalT	5.286568e-91	3.171941e-90	3.171941e-90	156.7702
	SiaT	1.723366e-03	1.034020e-02	2.068040e-03	245.7453

Table S12. Pearson correlation coefficients between experimental glycan peaks [27] and estimated total concentrations of enzymes in the Korčula (*n* = 915) and Vis (*n* = 890) populations

Glycan peaks	Korčula						Vis					
	**FucT**	**GnT I**	**GnT II**	**GnT III**	**GalT**	**SiaT**	**FucT**	**GnT I**	**GnT II**	**GnT III**	**GalT**	**SiaT**
GP_1	-0.30	-0.09	-0.71	-0.09	-0.15	0.04	-0.13	-0.11	-0.58	-0.04	-0.17	-0.09
GP_2	-0.36	-0.07	-0.18	0.24	-0.43	-0.36	-0.48	-0.13	-0.23	0.24	-0.49	-0.41
GP_4	0.07	0.31	-0.24	0.00	**-0.93**	-0.32	-0.02	0.26	-0.33	-0.02	**-0.94**	-0.29
GP_5	-0.40	-0.65	-0.12	0.04	0.01	0.31	-0.34	-0.65	-0.13	0.07	0.00	0.07
GP_6	-0.13	0.06	-0.23	0.56	-0.80	-0.51	-0.24	0.02	-0.30	0.51	-0.83	-0.50
GP_7	-0.46	-0.25	-0.28	0.09	-0.03	-0.14	-0.52	-0.31	-0.18	0.25	-0.07	-0.23
GP_8	0.57	0.28	0.46	-0.07	0.19	-0.31	0.46	0.25	0.39	-0.12	0.30	-0.20
GP_9	0.45	0.14	0.12	-0.24	-0.08	-0.24	0.42	0.12	0.14	-0.33	0.01	-0.09
GP_{10}	0.16	-0.02	0.16	0.80	-0.04	-0.44	0.03	-0.07	0.11	0.75	0.00	-0.45
GP_{11}	-0.26	-0.33	-0.15	0.47	-0.24	0.06	-0.16	-0.23	-0.14	0.51	-0.28	-0.15
GP_{12}	-0.33	-0.32	-0.10	0.01	0.40	-0.14	-0.34	-0.32	-0.03	0.11	0.35	-0.18
GP_{13}	-0.21	-0.25	-0.15	0.22	0.19	-0.20	-0.20	-0.27	-0.18	0.25	0.16	-0.11
GP_{14}	0.39	-0.14	0.36	-0.27	**0.91**	-0.01	0.35	-0.15	0.33	-0.26	**0.93**	0.06
GP_{15}	-0.01	-0.26	0.07	0.31	0.64	-0.09	0.17	-0.26	0.18	0.28	0.72	-0.08
GP_{16}	0.12	-0.04	-0.06	-0.10	0.05	0.36	0.12	-0.09	0.00	0.01	0.04	0.45
GP_{17}	-0.73	-0.13	-0.28	-0.03	0.05	0.30	-0.67	-0.16	-0.11	-0.03	0.03	0.19
GP_{18}	0.31	-0.19	0.33	-0.29	**0.93**	0.26	0.34	-0.18	0.33	-0.26	**0.95**	0.33
GP_{19}	0.08	-0.16	0.18	0.40	0.09	0.29	0.08	-0.02	0.05	0.33	0.09	0.38
GP_{21}	-0.76	-0.20	-0.27	-0.03	0.07	0.66	-0.70	-0.14	-0.07	0.05	0.08	0.49
GP_{22}	-0.29	-0.21	-0.01	0.41	0.08	0.20	-0.51	-0.21	-0.07	0.35	0.08	0.28
GP_{23}	0.07	-0.07	0.18	-0.39	0.57	0.78	0.25	0.04	0.18	-0.35	0.54	0.81
GP_{24}	0.12	-0.05	0.25	0.41	0.20	0.51	0.13	0.06	0.11	0.33	0.18	0.64

Table S13. Pearson correlation coefficients between experimental glycan peaks [27] and age of individuals in the Korčula (n = 915) and Vis (n = 890) populations

**Glycan peaks**	**Korčula**	**Vis**
GP_1	0.06	0.03
GP_2	0.23	0.28
GP_4	0.54	0.56
GP_5	0.01	-0.01
GP_6	0.52	0.52
GP_7	-0.05	0.02
GP_8	-0.21	-0.25
GP_9	0.01	-0.07
GP_{10}	0.14	0.04
GP_{11}	0.24	0.21
GP_{12}	-0.31	-0.25
GP_{13}	-0.15	-0.07
GP_{14}	**-0.62**	**-0.63**
GP_{15}	-0.31	-0.42
GP_{16}	0.10	0.06
GP_{17}	0.04	0.05
GP_{18}	-0.56	-0.59
GP_{19}	0.03	0.06
GP_{21}	-0.02	0.02
GP_{22}	0.04	0.02
GP_{23}	-0.32	-0.25
GP_{24}	0.04	0.05

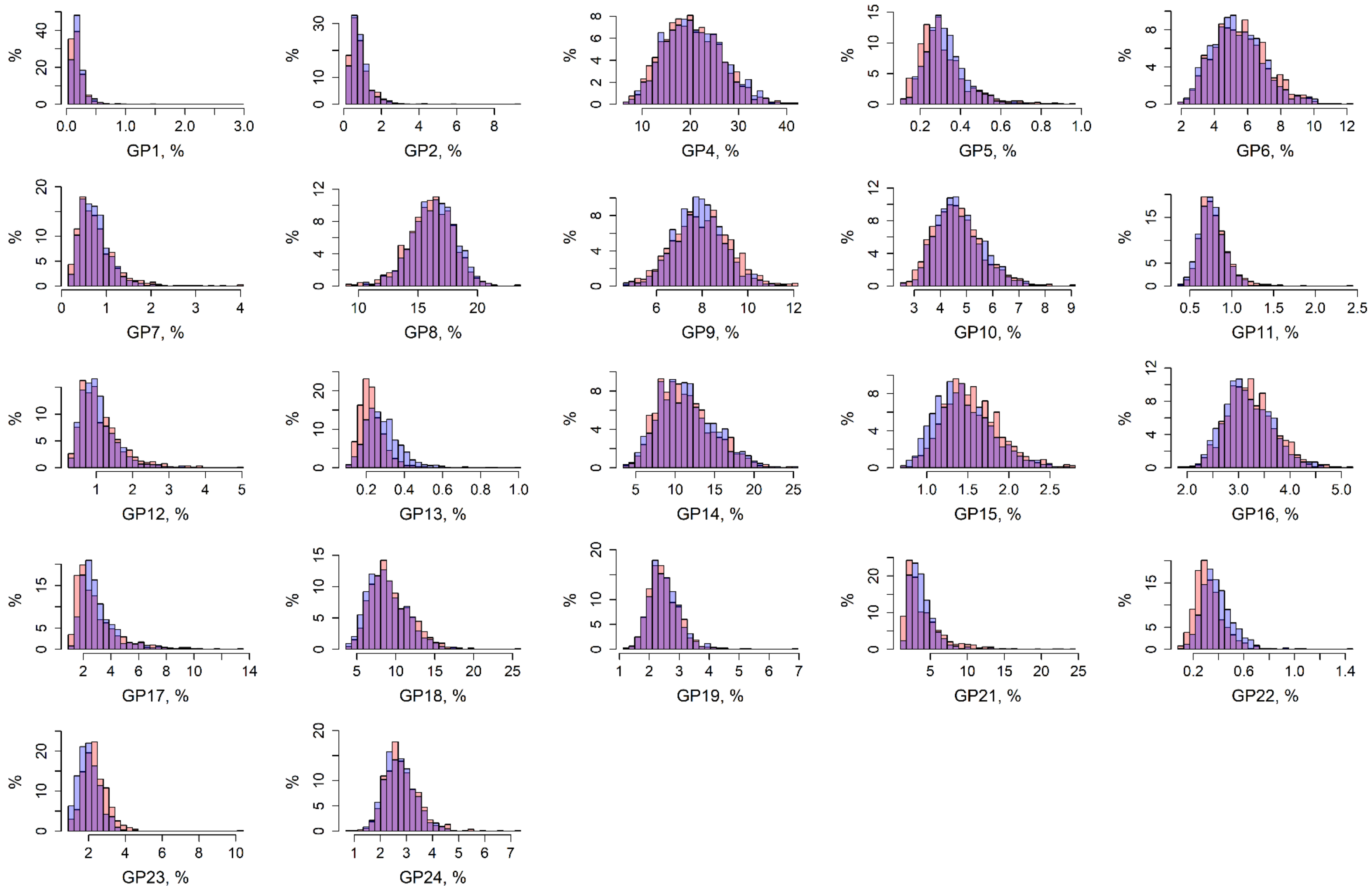

Figure S2. Distribution of experimental glycan peaks [27] for individuals from Korčula (red, *n* = 915) and Vis (blue, *n* = 890) populations. The overlapping regions are shown in purple.
